# Abnormal Rich Club Organization of Structural Network as a Neuroimaging Feature in Relation With the Severity of Primary Insomnia

**DOI:** 10.3389/fpsyt.2020.00308

**Published:** 2020-04-23

**Authors:** Yunfan Wu, Zhihua Zhou, Shishun Fu, Shaoqing Zeng, Xiaofen Ma, Jin Fang, Ning Yang, Chao Li, Yi Yin, Kelei Hua, Mengchen Liu, Guomin Li, Kanghui Yu, Guihua Jiang

**Affiliations:** ^1^ Department of Medical Imaging, Guangdong Second Provincial General Hospital, Guangzhou, China; ^2^ Department of Neurology, The First Affiliated Hospital, School of Clinical Medicine of Guangdong Pharmaceutical University, Guangzhou, China; ^3^ The Second School of Clinical Medicine, Southern Medical University, Guangdong Second Provincial General Hospital, Guangzhou, China

**Keywords:** primary insomnia, diffusion tensor imaging, human connectome, rich club, feature

## Abstract

**Purpose:**

Insomnia is the most prevalent sleep complaint in the general population but is often intractable due to uncertainty regarding the underlying pathomechanisms. Sleep is regulated by a network of neural structures interconnected with the core nodes of the brain connectome referred to as the “rich club”. We examined alterations in brain rich-club organization as revealed by diffusion tensor imaging (DTI) and the statistical relationships between abnormalities in rich-club metrics and the clinical features of primary insomnia (PI).

**Methods:**

This study recruited 43 primary insomnia (PI) patients and 42 age-, sex-, and education level-matched healthy controls (HCs). Differences in global and regional network parameters between PI and healthy control groups were compared by nonparametric tests, and Spearman's correlations were calculated to assess associations of these network metrics with PI-related clinical features, including disease duration and scores on the Pittsburgh Sleep Quality Index, Insomnia Severity Index, Self-Rating Anxiety Scale, and Self-Rating Depression Scale.

**Results:**

Weighted white matter networks exhibited weaker rich-club organization in PI patients than HCs across different thresholds (50%, 75%, and 90%) and parcellation schemes [automated anatomical labeling (AAL)-90 and AAL-1024]. Aberrant rich-club organization was found mainly in limbic-cortical-basal ganglia circuits and the default-mode network.

**Conclusions:**

Abnormal rich-club metrics are a characteristic feature of PI-related to disease severity. These metrics provide potential clues to PI pathogenesis and may be useful as diagnostic markers and for assessment of treatment response.

## Introduction

Insomnia is defined as trouble falling or staying asleep accompanied by subjective complaints of poor sleep quality and impaired daytime functioning (1). Insomnia is the most prevalent sleep disorder in the general population (2). Insomnia not only disrupts the normal circadian rhythm and sleep cycle but also profoundly affects health and aging (3). Indeed, there is mounting evidence suggesting that circadian clock disturbances and insomnia impair cognition, particularly in older adults, and even increase the risks of neurodegenerative disorders (3), such as Alzheimer's disease (AD) and Parkinson's disease. Primary insomnia (PI) defined as insomnia not attributable to other disorders. Despite high prevalence and a substantial socioeconomic burden, the underlying pathophysiological mechanisms of PI remain elusive (1, 4).

Neuroimaging studies (5–7) have implicated widespread brain regions in the pathophysiology of PI, including the hippocampus, insula, thalamus, amygdala, caudate nucleus, and prefrontal gyrus. Collectively, these studies suggest that PI arises from network dysfunction rather than dysfunction of a single isolated brain region. Combined diffusion tensor imaging (DTI) tractography and graph theory have been applied successfully to identify the network abnormalities associated with the psychopathology of AD (8) and schizophrenia (9). In our previous research, brain network analysis using 1.5T magnetic resonance imaging can be used to elucidate the pathophysiology of PI (10). However, studies on the brain connectome have identified a core network of highly interconnected neocortical nodes essential for global integration, referred to as the “rich club” (11). Rich-club nodes (hubs) appear preferentially vulnerable to neurodegenerative diseases compared to non-hub nodes (9, 12), suggesting possible relevance to PI onset or severity. Therefore, we examined if the PI-related white matter (WM) network revealed by DTI tractography exhibits altered rich-club organization. In addition to elucidating the pathogenesis of PI, such information could identify neuroimaging markers for diagnosis and treatment development. However, the alterations of rich club organization in PI patients are still unclear.

We hypothesized that the PI-related brain WM network would have the alteration of rich club organization by DTI tractography by 3.0MR scanner. This study will increase our understanding of PI-related pathophysiology. Moreover, we hope to explore a more stable imaging feature in the structural network, which is of great significance for clinical diagnosis and evaluation prognosis of PI.

## Materials and Methods

This prospective study was approved by the ethics committee of Guangdong Second Provincial General Hospital. Written informed consent was obtained from each participant.

### Patients and Healthy Control Subjects

Between April 2014 and April 2016, 85 right-handed individuals, comprising 43 PI patients and 42 age-, sex-, and education level matched healthy control (HC) subjects, were finally recruited in this study. The diagnosis was performed by two specialized neurologists with 15 years of experience. The diagnosis criteria for PI patients was based on the criteria of Diagnostic and Statistical Manual of Mental Disorders, version 5 (DSM-V) (13): (a) self-complaint of more than three months with maintaining sleep, difficulty falling asleep, or early awakening; (b) no other sleep disorders (parasomnia, hypersomnia, or sleep-related movement disorder); (c) insomnia caused by no serious organic disease or no severe mental disease, such as brain stroke, depression (SDS < 70), and anxiety (SAS < 70); (d) dextromanuality evaluated by the Edinburgh Handedness Inventory (EHI) criteria; (e) age of 18–60 years. Moreover, all PI patients were recruited at the time of initial diagnosis, or required not have taken medicine 2 weeks before experiment to prevent drug effects. In addition, HC subjects were required to meet the following criteria: (a) the age-, hand dominant-, and gender-matched participants from the local community; (b) good sleep quality and the Insomnia Severity Index (ISI) score < 7 or the Pittsburgh Sleep Quality Index (PSQI) score < 7. The exclusion criteria for all subjects were as follows: (a) an abnormal signal as verified by conventional T1- or T2-weighted fluid-attenuated inversion recovery MR imaging; (b) pregnant; (c) severe brain lesions detected by MR.

Each participant underwent a standardized clinical evaluation protocol before MR. The neuropsychological tests were: (i) PSQI and ISI determination for sleep quality evaluation; (ii) Self-Rating Anxiety Scale (SAS) and Self-Rating Depression Scale (SDS) score determination for estimating the mental status of PI patients.

### MR Acquisition

MR images for all subjects were acquired on a Philips 3.0 T MR system (Achieva Nova-Dual; Best, Netherlands). Each participant was placed in the supine position with eyes closed and the head snugly restricted by a belt and foam pads. T1-weighted images were acquired with the following parameters: TR, 8 ms; TE, 4 ms; matrix, 256×256; FOV, 250×250 mm; flip angle, 8°; section thickness, 1 mm. DTI imaging was acquired with the following parameters: TR, 10,000 ms; TE, 101 ms; matrix, 128×128; FOV, 224×224 mm; flip angle, 90°; slices = 69; section thickness, 2.2 mm; 32 independent, non-collinear diffusion weighting gradient direction b-value 1,000 sec/mm^2^ and one additional image without diffusion weighting (b = 0 sec/mm^2^). Two radiologists more than 15 years of experience reviewed and verified these images to ensure segmentation quality.

### Data Preprocessing and Network Construction

All raw DTI data were preprocessed by the Pipeline for Analyzing Brain Diffusion Images toolkit (PANDA) software (www.nitrc.org/projects/panda). The detailed procedures of the PANDA toolbox were described by Cui Z et al. (14). Ninety network nodes were defined by the automated anatomical labeling (AAL) atlas (15) without the cerebellum (45 for each hemisphere, **Table 1**). Deterministic tractography was constructed WM networks for each subject with the Fiber Assignment by Continuous Tracking algorithm (16). The WM deterministic fiber tracking was terminated when a tracking turn angle was above 45° or the streamline reached a voxel with an fractional anisotropy (FA) < 0.2 between two consecutive voxels (17). We defined the weight of each effective edge between two nodes (i and j) of the WM structure as the product of FN and mean FA along the fiber bundles, and normalized by the average volume of two connecting nodes (w_i__j_ = FN * FA/volume) as previous studies described (18, 19). Finally, a weighted matrix of 90 × 90 structural network was constructed for each subject.

**Table 1:** ALL-90 templates.

Regions abbreviations	Brain Regions	Regions abbreviations	Brain regions
PreCG	Precentral gyrus	LING	Lingual gyrus
SFGdor	Superior frontal gyrus, dorsolateral	SOG	Superior occipital gyrus
ORBsup	Superior frontal gyrus, orbital part	MOG	Middle occipital gyrus
MFG	Middle frontal gyrus	IOG	Inferior occipital gyrus
ORBmid	Middle frontal gyrus, orbital part	FFG	Fusiform gyrus
IFGoperc	Inferior frontal gyrus, opercular part	PoCG	Postcentral gyrus
IFGtriang	Inferior frontal gyrus, triangular part	SPG	Superior parietal gyrus
ORBinf	Inferior frontal gyrus, orbital part	IPL	Inferior parietal, but supramarginal and angular gyrus
ROL	Rolandic operculum	SMG	Supramarginal gyrus
SMA	Supplementary motor area	ANG	Angular gyrus
OLF	Olfactory cortex	PCUN	Precuneus
SFGmed	Superior frontal gyrus, medial	PCL	Paracentral lobule
ORBsupmed	Superior frontal gyrus, medial orbital	CAU	Caudate nucleus
REC	Gyrus rectus	PUT	Lenticular nucleus, putamen
INS	Insula	PAL	Lenticular nucleus, pallidum
ACG	Anterior cingulate and paracingulate gyrus	THA	Thalamus
DCG	Median cingulate and paracingulate gyrus	HES	Heschl gyrus
PCG	Posterior cingulate gyrus	STG	Superior temporal gyrus
HIP	Hippocampus	TPOsup	Temporal pole: superior temporal gyrus
PHG	Parahippocampal gyrus	MTG	Middle temporal gyrus
AMYG	Amygdala	TPOmid	Temporal pole: middle temporal gyrus
CAL	Calcarine fissure and surrounding cortex	ITG	Inferior temporal gyrus
CUN	Cuneus		

### Network Analysis

All global and nodal metrics were calculated using the GRETNA toolbox (Release 2.0.0) (http://www.nitrc.org/projects/gretna) (20), including the following graph metrics: small-world coefficient (sigma), clustering coefficient (Cp), characteristic path length (Lp), normalized Cp (gamma), normalized Lp (lambda), global efficiency (Eg), local efficiency (Eloc), and nodal efficiency. These metrics were thoroughly defined previously by Rubinov and Sporns (21). We generated a receiver operating characteristic curve (AUC) over the sparsity threshold range of 0.10–0.40 with an interval of 0.01 to discriminate between-group differences with 1,000 matched random networks.

As described in previous studies (11, 22), a group-average connection matrix was defined as effective when it was found in at least 75% of the subjects in this group. The high rich-club coefficient (Φ^w^) measures the connection ability between a group of nodes with larger than the k degree. And the k degree was defined as the number of each node's binary connections (9) by computing the population of 1,000 random networks. The value of K degree in this study was 7. Weighted network edges were classified into three categories, including rich club connections (linking non-rich club regions to rich ones), feeder connections (linking rich club regions to non-rich ones), and local connections (linking non-rich club regions) connections. By 10,000 nonparametric permutation tests and double tail p value by Holm-Bonferroni (FWE) Correction, the connection strengths of rich club, feeder, and local connections were compared between the two groups. All nodes visualized with the BrainNet Viewer software (http://www.nitrc.org/projects/bnv/).

### Statistical Analysis

The SPSS 16.0 software (SPSS Inc., Chicago, USA) was used to compare demographic and clinical characteristics. Normality was tested with the Shapiro-Wilk test. Education time, age, PSQI, ISI, SAS, and SDS scores in PI and HC participants were assessed by the Mann-Whitney U test. The qualitative variable of sex was compared by a χ2 test. Ten thousand nonparametric permutation tests were performed to determine between-group differences in global and nodal network metrics. After between-group differences of network metrics were identified in the above metrics, Spearman's correlations were performed for associations of various metrics with clinical scores in PI patients. All correlation tests removed age, gender, and education level as covariates. Holm-Bonferroni Correction (FWE) was performed to address the multiple comparisons at a significance level of 0.05 (p < 0.05). All statistical analyses were performed using the MATLAB 2016 software (Matlab, MathWorks, USA) and SPSS 16.0 software.

### Reproducibility Analysis

We found the application of different thresholds (FN ≥ 1, 2, 3, 5, or 10) in AAL-90 templates and a threshold (FN = 1) in different templates (AAL-1024 and HOA-1024) had little effect on the results (**Table 2** and **Figure 5**).

**Table 2:** Group Differences in Global Network Metrics.

Network Metrics	p-value						
	W i j =FA*FN/Volume						
	ALL-90					HOA-1024	ALL-1024
	FN≥1	FN≥2	FN≥3	FN≥5	FN≥10	FN≥1	FN≥1
*σ*	<0.001	<0.001	<0.001	<0.001	<0.001	<0.001	<0.001
γ	<0.001	<0.001	<0.001	<0.001	<0.001	<0.001	<0.001
λ	n.s.	n.s.	n.s.	n.s.	n.s.	n.s.	n.s.
Cp	<0.001	<0.001	<0.001	<0.001	<0.001	0.001	0.003
Lp	n.s.	n.s.	n.s.	n.s.	n.s.	0.011	0.022
Eg	0.013	0.014	0.012	0.012	0.011	0.009	0.020
Eloc	0.016	0.015	0.017	0.018	0.017	n.s.	n.s.

The structural networks were construct by using the definitions of W _i__j_ =FA*FN / Volume. The threshold of FN ≥ 1, or FN ≥ 2, or FN ≥ 3, or FN ≥ 5, or FN ≥ 10 indicates that two regions were connected if at least 1 or 2 or 3 or 5 or 10 streamlines exist between a pair of brain regions. The high spatial resolution template (HOA-1024 and ALL-1024) was also used in constructing the structural networks. The comparisons of network metrics between groups were performed by using a nonparametric permutation test while removing the effects of age, sex, and years of education. (P< 0.05; Benjamin-Hochberg FDR corrected) indicated a significant group difference.

FA, fractional anisotropy; FN, streamline number; σ, small-worldness; γ, normalized clustering coefficient; λ, normalized shortest path length; Cp, cluster coefficient; Lp, characteristic path length; Eg, global efficiency; Eloc, local efficiency; n.s., non-significant.

## Results

### Demographic and Clinical Characteristics

Age, sex, and years of education did not significantly differ between the PI and HC groups (all P > 0.05). Meanwhile, PSQI, ISI, SAS, and SDS scores were significantly higher in the PI group compared with the HC group (**Table 3**).

**Table 3:** Demographics and Clinical Characteristics of All Participants.

Characteristic	PI participants (n = 43)	HC participants (n = 42)	P value
Age (years)	41.29± 11.36	39.42± 9.33	0.30
Sex (male/female)	21/22	17/25	0.51
Duration of education (yeas)	8.02± 3.37	7.63 ± 4.59	0.58
PSQI	16.32± 3.76	2.55± 2.47	<0.001
ISI	20.72± 3.75	2.63± 2.65	<0.001
SAS	47.66 ± 8.59	5.91± 10.87	<0.001
SDS	52.40 ± 8.75	6.97 ± 11.97	<0.001

Unless otherwise noted, data are expressed as mean ± SD.

PIQS, Pittsburgh Sleep Quality Index; ISI, Insomnia Severity Index; SAS, Self-Rating Anxiety Scale; SDS, Self-Rating Depression Scale; PI, insomnia; HC, healthy control.

### Global Topology of WM Structural Networks

**Figure 1:**
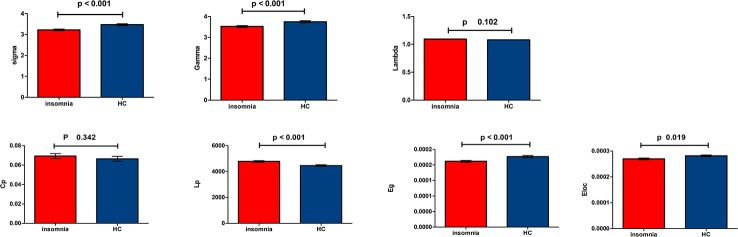
Bar chart show differences in global topologic properties of the white matter (WM) structural networks between primary insomnia patients and healthy control (HC) group. Bars and error bars represent mean values and standard deviations, respectively. After removing the effects of age, sex, and years of education, significant differences were found in sigma (P < 0.001), gamma (P < 0.001), Eg (P < 0.001), and Eloc (P = 0.019) in patients with primary insomnia. sigma, small-worldness; gamma, normalized clustering coefficient; Lp, characteristic path length; lambda, normalized Lp; Cp, clustering coefficient; Eg, global efficiency; Eloc, local efficiency; PI, primary insomnia; HC, healthy control.

All WM structural networks showed characteristic small-world properties across the selected thresholds (gamma > 1, lambda ≈ 1, and sigma > 1). Compared with HC controls, PI patients exhibited significantly reduced sigma (P < 0.001), global efficiency (P < 0.001), and local efficiency (P = 0.019) (**Figure 1**). The application of different thresholds and AAL templates had little effect on the results (**Table 2**).

### Rich Club Organization

**Figure 2:**
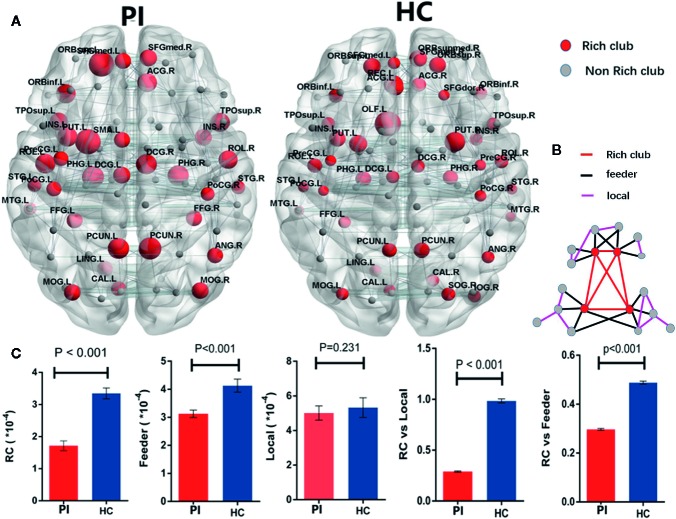
Rich - club regions and connections. **(A)** The figure shows the rich club regions of the WM networks in the primary insomnia and HC control groups. Sizes of rich club node reflect their nodal connection strength and colored to indicate rich - club regions (red) or non - rich - club regions (gray). The networks of rich - club regions were constructed by the WM backbone of all the participants in each group with a group - level threshold of 75% (ALL - 90). **(B)** Figure B represents a schematic diagram that all edges of brain structural networks are divided into 3 distinct classes and colored to indicate rich club connections (red), feeder connections (blue), and local connections (green). **(C)** The bars and error bars represent group differences in the rich - club, feeder, and local connection strengths, while removing the effects of age, sex, and years of education, PI, primary insomnia; HC, healthy control.

According by previous studies (9, 11), rich club organizations were identified in structural networks from WM by averaging—group matrix in all participants of each group with a group—level threshold of 75%. For each group, rich club regions were found as the top 32 high-degree brain regions, and 29 identical rich club distributions were found in the PI and HC groups (**Figure 2**).

Group analysis indicated significant group differences in rich club connections (P < 0.001) and feeder connections (P < 0.001), but not in local connections (P = 0.231) in PI patients compared with HCs. There is a significant decrease in rich club connections and feeder connections (nonparametric permutation tests). Across all individual datasets for WM construction, we identified a high consistency with group-level networks in rich club (P < 0.001) and feeder (P < 0.001) connections, consistent with previous study (9). Besides, we revealed a significantly lower proportion of connections between rich club and feeder connections (rich club/feeder, P < 0.001) and between rich club and local connections (rich club/local, P < 0.001) in PI and HC groups (**Figure 2C**). The application of different thresholds and AAL templates had little effect on the results (**Figure 5**).

### Associations of Network Metrics With Clinical Characteristics

**Figure 3:**
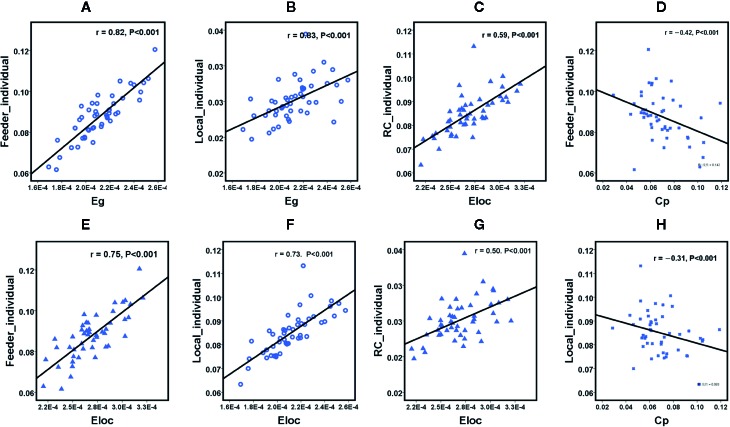
Scatterplot shows the relationships between rich club parameters and small world network characteristics in participants with primary insomnia ( A-H, respectively). PI = primary insomnia, HC = healthy control, RC= rich club connections, Local = local connections, Feeder= local connections, Cp = cluster coefficient, Eg = global efficiency, Eloc = local efficiency.

**Figure 4:**
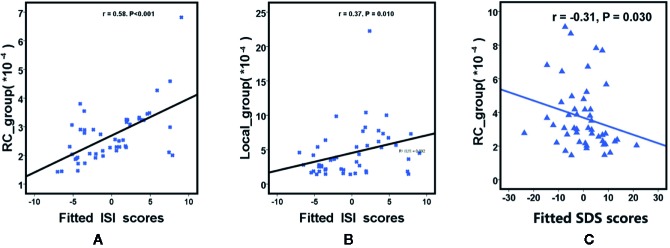
Scatterplot shows the relationships between rich club parameters (**A** is RC and **B** is Local ) and ISI scores in participants with primary insomnia **(A, B)**. Scatterplot shows the relationships between SDS scores and rich club connections (RC) in primary insomnia **(C)**. The fitted values indicate the residuals of Pittsburgh Sleep Quality Index scores, insomnia severity index scores and self - Rating depression scale scores after removing the effects of age, sex, and years of education. SDS= Self - Rating Depression Scale score, RC= rich club connections, local = local connections, ISI = insomnia severity index.

We further assessed the associations of other network metrics with clinical characteristics. Significant correlations were identified between global metrics (Eg, Eloc, Cp) and rich club metrics (**Figure 3**). Moreover, positive correlations were observed between ISI score and rich club connections (r = 0.58, P < 0.001), and between ISI scores and local connections (r = 0.37, P = 0.010), respectively (**Figure 3**). Meanwhile, a significantly negative correlation was found between rich club connections and SDS score (r = −0.31, P = 0.030) in the patient population (**Figure 4**). The fitted values indicated the associations remained after removing the effects of age, sex, and years of education.

**Figure 5:**
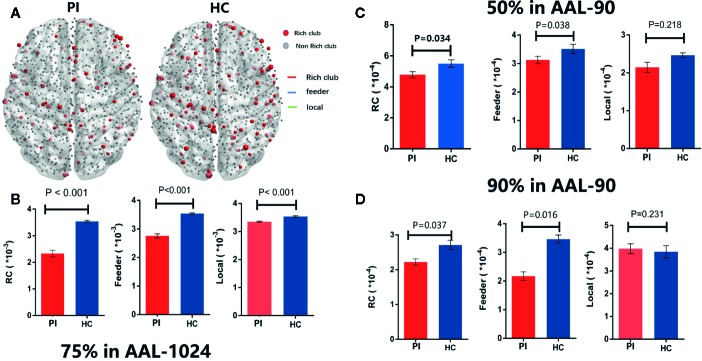
Rich - club regions and connections with different group - level thresholds. **(A)** The figure shows the rich club regions of the WM networks in the insomnia and HC control groups with75% group - level in ALL1024 templates. **(B)** The bars and error bars represent group differences in the rich - club, feeder, and local connection strengths with75% group - level in ALL - 1024, while removing the effects of age, sex, and years of education. Similarly, **(C)** and **(D)** represent the bars and error bars group differences in the rich - club, feeder, and local connection strengths with 50% (C) and 90% (D) group - level in ALL1024, respectively. PI= primary insomnia, HC = healthy control.

## Discussion

The main finding of this study is that PI patients exhibited weaker rich-club network metrics than matched HCs, primarily in limbic-cortical-basal ganglia circuits and the default-mode network (DMN). Furthermore, significant correlations were identified between insomnia severity (ISI score) and rich-club organization metrics. These findings were replicated with different grouping thresholds and parcellation schemes, suggesting that altered rich-club metrics are a relatively stable feature of PI suitable for early detection and assessment of disease severity.

Compared to matched controls, PI patients frequently demonstrate chronic attention impairments, working memory deficits and higher incidences of anxiety and depression (1). Fronto-limbic rich-club regions, including prefrontal cortex, insula, basal ganglia, parahippocampal cortex, and cingulate cortex, are major centers of cognition and emotion (23, 24). Prefrontal cortex (including the orbitofrontal cortex) also contributes to normal sleep physiology, dreaming (25, 26), consolidation of memory during sleep, and working memory (27), so dysfunction could disrupt sleep as well as daytime cognitive and emotional functioning. Insular abnormalities may also affect cognition and emotional regulation by influencing the activity of numerous other cognitive- and emotion-related regions (7). The lenticular nucleus and putamen are components of the basal ganglia with strong connections to the fronto-limbic system (28). The parahippocampal region plays an important role in the encoding and recognition of environmental scenes (29), and so will consolidate environmental and internal associations with insomnia, including the negative emotions caused by insomnia. Dysfunctions of the cingulate cortex and prefrontal cortex are associated with sustained sleep difficulties and cognitive impairment in insomnia (30). Thus, these alterations in rich-club organization, particularly in fronto-limbic circuits, likely contribute to the cognitive deficits and maladaptive emotions associated with PI. In addition to limbic-cortical-basal ganglia circuits, alterations in connectivity were also found among rich-club nodes within the DMN (e.g. praecuneus, prefrontal cortex, superior parietal lobule, cingulate cortex, temporal lobe, occipital lobe). According to Suh et al. (30) malfunctioning of the DMN during the wake-to-sleep transition may underlie sustained sleep difficulties and cognitive impairments in insomnia. Moreover, previous studies (31, 32) suggested the DMN dysfunction may be reflected by the heightened sensitivity and self-awareness in the dreams of PI patients. Therefore, our 3.0T DTI findings of altered rich-club organization within the PI-associated WM network, mainly limbic-cortical-basal ganglia circuits and DMN hubs, are in accordance with the typical symptomology of PI. Moreover, our findings revealed significant correlations between abnormal rich-club connectivity metrics and SDS scores, consistent with the strong associations among insomnia, depression, and poor global cognition (33).

Rich-club and feeder connections were significantly weaker in the PI group across different group thresholds (50%, 75%, and 90%) and parcellation schemes (AAL-90 and AAL-1024), underscoring the robustness of these observations. We speculate that PI may result from disruptions of WM connectivity among rich-club hubs, because the WM networks of all patients constructed by FA and FN. Collin et al. suggested a direct relationship between lower FA and reduced WM connectivity (34). He found that the FA of WM tracts among rich-club nodes increased with myelination during brain development. Thus, we speculate that rich-club nodes may become non-hubs during PI progression due to demyelination.

On the other hand, the global topologies of the WM network in PI patients exhibited significantly lower Eg and Eloc, and rich-club regions partially overlapped with the lower node efficiency region, especially the left insula (INS.L), anterior cingulate gyrus (ACG), (ROL.L), and right dorsal cingulate gyrus (DCG.R). Eg is a measure of functional integration and Eloc represents a measure of functional segregation (35). In addition, previous reports (9, 11, 12, 36) have provided evidence that rich-club connections are more vulnerable to neurodegenerative diseases than non-hub nodes. Thus, we speculate that disruption of rich-club organization reflects the impact of PI on integration and segregation of small-world networks, especially in the early disease period. This speculation is further supported by significant correlations between rich-club organization metrics and both Eg and Eloc.

Meanwhile in the latest decade, many studies have implicated abnormal connections among rich-club hubs in the pathophysiology of diseases such as AD (8) and schizophrenia (11). In the current study, rich-club metrics were positively correlated with PI severity (ISI score), strongly suggesting that disrupted connectivity among these nodes directly contributes to PI symptoms. Therefore, rich-club metrics may be useful network-based biomarkers for early PI detection and for assessing the severity of network damage. The relationships between pathologic alterations and the three distinct classes of rich-club connectivity in PI warrant further investigation.

### Limitations

Limitations of this study include no quantitative assessment of cognitive dysfunction and small sample size. In addition, the diagnosis of PI in the study is lack of polysomnography to exclude somatic diagnoses. Moreover, WM networks were constructed from DTI data using the deterministic fiber-tracking algorithm. However, deterministic tractography is not optimal for estimating crossing fibers, which may affect the accuracy of streamline count.

## Conclusion

This is the first report of abnormal rich-club organization in PI. Abnormal rich-club metrics are a characteristic manifestation of abnormal WM network structure in PI patients. Our results also suggest that rich-club metrics may be clinically relevant to the underlying pathogenesis and symptoms of PI and useful as biomarkers for the early detection and assessment of PI severity.

## Data Availability Statement

All datasets generated for this study are included in the article.

## Ethics Statement

The studies involving human participants were reviewed and approved by the Guangdong Second Provincial General Hospital Human Research Ethics Committee. The patients/participants provided their written informed consent to participate in this study.

## Author Contributions

YW and GJ conceived and designed the experiments. YW, SF, YY, KH, ML, and GL acquired the data. SZ, XM, JF, and KY performed the clinical data, which YW, CL, and NY analyzed. YW and ZZ wrote the article, which all authors reviewed and approved for submission.

## Funding

Supported by the National Natural Science Foundation of China (grant No. 81901729, U1903120, 81771807), the Natural Science Foundation of Guangdong (grant No.2015A030313723, 2019A1515011314), and the Science and Technology Planning Project of Guangdong (2016A020215125, 2017A020215077).

## Conflict of Interest

The authors declare that the research was conducted in the absence of any commercial or financial relationships that could be construed as a potential conflict of interest.

## Abbreviations

PI, primary insomnia; HC, healthy control; WM, white matter; DTI, diffusion tensor imaging; FA, fractional anisotropy; FN, streamline number.
